# Recovery of the soil fungal microbiome after steam disinfection to manage the plant pathogen *Fusarium solani*

**DOI:** 10.3389/fpls.2023.1128518

**Published:** 2023-04-21

**Authors:** Eric R. Larson, Sharifa G. Crandall

**Affiliations:** ^1^ Department of Plant Pathology and Environmental Microbiology, Pennsylvania State University, University Park, PA, United States; ^2^ Microbiome Center, Penn State Institutes of the Life Sciences, University Park, PA, United States

**Keywords:** *Trichoderma* (*Trichoderma harzianum*), soil health, management of plant pathogens, soil amedments, soybean, microbial communities, fungi, microbiome

## Abstract

Soil disinfection using high temperatures *via* steam is a promising approach to manage plant pathogens, pests, and weeds. Soil steaming is a viable option for growers who are moving away from dependence on chemical soil fumigants, especially in plant nursery or high tunnel environments. However, there are few studies that investigate how soil steaming causes substantial disturbance to the soil by killing both target pathogens and other soil biota. Steaming treatments also change the trajectory of the soil microbiome as it reassembles over time. Growers are interested in the health of soils after using steam-disinfection, especially if a virulent pathogen colonizes the soil and then flourishes in a situation where there are very few microbes to suppress its growth. Should recruitment of a virulent pathogen occur in the soil, this could have devasting effects on seed germination, seedling establishment and survival. Beneficial microbes are often used to prevent the colonization of plant pathogens, especially after a soil-steaming event. Here, we experimentally test how soil fungal communities assemble after steaming disinfection. We introduce to steam-treated soil *Fusarium solani*, an important fungal pathogen of soybean and *Trichoderma harzianum*, a known beneficial fungus used for soilborne pathogen suppression. Results show that *F. solani* significantly affects the relative abundance and diversity of the soil fungal microbiome, however, *T. harzianum* does not mitigate the amount of *F. solani* in the steam treated soil. Within the *T. harzianum* microbial addition, the soil fungal communities were similar to the control (steaming only). This result suggests inoculating the soil with *T. harzianum* does not drastically alter the assembly trajectory of the soil fungal microbiome. Other soil amendments such as a combination of *Trichoderma* spp. or other genera could suppress *F. solani* growth and shift soil microbiome composition and function post-steaming, however, more experimental research is needed.

## Introduction

Plant pathogens possess the potential to devastate crops and forests worldwide (Agrios, 2005; Dart and Chastagner, 2007). In agricultural systems, plant pathogens decrease crop yield, reduce marketability, and in some cases, cause complete crop loss which in turn impacts both domestic and global food security (Strange and Scott, 2005). The Food and Agricultural Organization of the United Nations (FAO) estimates that $220 billion dollars are spent each year globally to manage plant diseases (FAO, 2022). Soilborne plant pathogens (e.g., fungi, oomycetes, bacteria, viruses and nematodes) are especially challenging to manage.

Management efforts for soilborne fungal and oomycete plant pathogens are often unsuccessful because many possess the ability to disperse through soil pores and persist for long periods of time in the soil spore bank (Panth et al., 2020). Soilborne pathogens can disperse between locations through the transfer of soil on farm machinery, from infected plant pots that are moved between plant nurseries or even between continents (Heungens et al., 2012; Parke et al., 2019; Frankel et al., 2020). Resting spores, such as chlamydospores or sclerotia, are structures that typically have a melaninzed or pigmented outer rind that protects them from both ultraviolet light and desiccation (Sumner, 1996; Panth et al., 2020). This adaptation allows fungal soilborne pathogens such as *Rhizoctonia solani* to remain in the spore bank for decades at a time if left unmanaged.

### Soil steam disinfection for pathogen management

Starting in the late 1880s in Germany, agronomists used steam to eliminate plant and animal pathogens found in soil (Mihajlović et al., 2017; Arancibia, 2020). The soil steaming method was introduced to the United States (US) in 1893 and for the first half of the 20^th^ century was predominately used for small scale production of high value crops such as tobacco, tomato, and greenhouse production of ornamentals (Johnson, 1946; Hansen et al., 2011; Arancibia, 2020). In the 1950s newly introduced chemical pesticides and fumigants became the main method for soilborne pathogen management (Wilhelm, 1966; Arancibia, 2020). Since the Montreal Protocol in 1987 there has been a slow yet steady phasing out of fumigants due to their broad toxicity and negative impacts on the environment and human health (Arancibia, 2020; Gambhir, 2020). The mandated disuse of methyl bromide for soilborne pathogen management in important crops resulted in an urgent need to find alternative, more sustainable phytosanitary approaches (Roux-Michollet et al., 2010; Samtani et al., 2012; Schweigkofler et al., 2014). Soil steaming disinfection kills pests and soilborne pathogens without the toxic side effects because water, without chemicals or other imputs, is turned into steam and applied to the soil at high temperatures (~70–90°C) (Katan, 2000; Fennimore and Goodhue, 2016; Arancibia, 2020). Recently, soil steaming for plant disease management has increased in demand and provides a promising management approach in agriculture (Atallah et al., 2011; Mihajlović et al., 2017). Steam is used to kill plant pathogens mainly in indoor settings, although the method is growing traction for use in high tunnel farming and is in the process of being tested for field crops (Schweigkofler et al., 2014; Arancibia, 2020). The American Plant Health Inspection Service (APHIS) reports steam disinfection as an official management option for treating soils in plant nurseries (USDA APHIS, 2020). The successful management of these pathogens lies at the intersection of agriculture, ecology, ecological restoration, and environmental remediation. There is an urgent need to understand the underlying science of how soil-steaming disinfection disturbs the soil microbiome and whether there are alternate trajectories for the microbial community diversity and persistence during soil recovery. Understanding these patterns and the underlying functions of these microbes that contribute to soil fertility (e.g., nitrogen and phosphorus cycling, organic matter) is critical for growers to improve soil and plant health.

### Ecological disturbance, soil microbiomes, and pathogen management

A major challenge today is to understand how certain ecological disturbances, both biotic and abiotic, can shift the composition and abundance of microbes that constitute the soil microbiome. Soil microbiomes are susceptible to disturbance events (Shade et al., 2012) and their recovery is known to be impacted largely by abiotic factors such as temperature, salinity, pH, or moisture (Shade et al., 2012; Thakur and Geisen, 2019; Kaminsky et al., 2021). It is known that biotic factors affect the microbiome, but the impact of microbial interactions on soil microbiome dynamics is far less studied (Thakur and Geisen, 2019). As the soil microbiome recovers post-disturbance, it can reach pre-disturbance dynamics or a new alternative stable state where microbial communities persist that are different from the pre-disturbance communities (Shade et al., 2012). Steaming for pathogen management shifts the composition and abundance of microbes; the question that follows is: will the recovering microbiome reach the pre-steam stable state or a new stable state, and can the community trajectory (abundance and diversity) be impacted by the early colonization of pathogenic and/or beneficial microbes?

Previous soil steaming research provides insight into post-steaming microbial recruitment patterns. Diversity in the bacterial microbiome spikes immediately post-steaming, then diversity and abundance levels off within 1–2 months (Crandall et al., 2022). However, the soil community composition doesn’t necessarily resemble pre-disturbance conditions. Certain communities of bacteria, such as Firmicutes, are most abundant 1–2 weeks post-steaming; this is not surprising as Firmicute endospores have been shown to survive other high temperature disturbances such as forest fires or autoclaving (Kaminsky et al., 2021; Fox et al., 2022). Soil fungal microbiomes, in contrast, exhibit a shift in composition and diversity post-disturbance with certain taxa blinking in and out of existence in the soil microbiome (Crandall et al., 2022). For this study we focus on fungal microbiome dynamics. Our rationale is: (1) a marked pattern of succession is found within the soil fungal microbiome as compared to bacterial microbiomes and (2) there are fewer studies that track fungal microbiome recovery.

This work examines the top-down effect that *Trichoderma harzianum* and *Fusarium solani* have on the soil microbiome when introduced post steaming. The majority of work on microbiome recovery after a disturbance focuses on the bottom-up regulation that plants have on soil microbe dynamics (Thakur and Geisen, 2019). Recent literature identifies microbial interactions as regulators of the soil microbiome structure, particularly the competition for resources (Bahram et al., 2018). Predation and competition can be key determinants of diversity in a community, especially when the competitor is a fast growing virulent (hereafter “aggressive”) plant pathogen (Roy, 1997; Roncero et al., 2003; Chesson and Kuang, 2008; Schmitz et al., 2010; Fukami, 2015). The objective for this research was to investigate the impact of an aggressive fungal pathogen and beneficial fungus on the soil fungal microbiome as it recovers post-steaming.

Our model organisms were the plant pathogen *Fusarium solani*, the cause of root rot in soybean (*Glycine max*) and *Trichoderma harzianum*, a known beneficial to soybean (Roy, 1997; Kabir et al., 2022). It had been shown that *Fusarium* spp. have an impact on the soil microbiome. Studies using fumigation to facilitate the removal of *Fusarium* spp. suggest that soil microbiomes were able to rebuild a resistance to *Fusarium* spp. post-fumigation. This indicates that the initial *Fusarium* spp. infections (prior to steaming) were suppressing the emergence of resistance to a pathogen (Shen et al., 2018; Xue et al., 2019; Deng et al., 2021; Zhang et al., 2021). There are few to no studies that quantify how plant pathogens like *F. solani* colonize the soil and influence microbiome recovery after a disinfestation event, such as steaming, *in-vivo.* There is also a growing body of literature that investigates the interaction between microorganisms used as biocontrols for soilborne pathogens (Sood et al., 2020). Specific fungi in the genus *Trichoderma* show antagonistic interactions toward common and virulent soil pathogens such as *F. solani* (Daniel and Filho, 2007; Pimentel et al., 2020; Sood et al., 2020; Bellini et al., 2023). *Trichoderma* spp. can elicit a bottom-up effect on the soil microbiome due to their positive interaction with plants, but the focus of this study was on the soil microbiome pre-planting. How microbes interact with one another is context dependent and within this study the microbiomes were assessed in the context of recovery post-steaming (Zhou et al., 2021).

We asked: how does the soil microbiome recover after a steaming event when the soil is inoculated with a fungal plant pathogen and a beneficial biocontrol fungus? Does the presence of these two fungi change the composition, diversity, and function of the soil fungal microbiome? We questioned if *T. harzianum*, in its capacity as a beneficial, would mitigate the effects of *F. solani*. We hypothesized that the early introduction of both fungi would significantly alter the development of the soil fungal microbiome after steaming. We expected that *T. harzianum* would suppress the growth of *F. solani* when added together. Our last question was: Is there a significant effect on the microbial community composition when a beneficial microbe is added to the soil in order to suppress a pathogen? Due to its capacity to parasitize *F. solani*, we hypothesized that *T. harzianum* would decrease the abundance of *F. solani* regardless of when it was applied. The usefulness of steam in managing soil pathogens has been validated through other research studies, (Atallah et al., 2011; Schweigkofler et al., 2014; Mihajlović et al., 2017), but the impacts on the microbial community remain unclear. Our work provides insight into how growers and land managers can best use beneficial fungi such as *T. harzianum* post-steaming. The purpose of this research is to provide baseline information in order to build predictive models to understand how the biotic composition of the soil can either facilitate or hinder the potential for plant germination, establishment, and survival.

## Methods

### Greenhouse cold frame experiment

**Figure 1:**
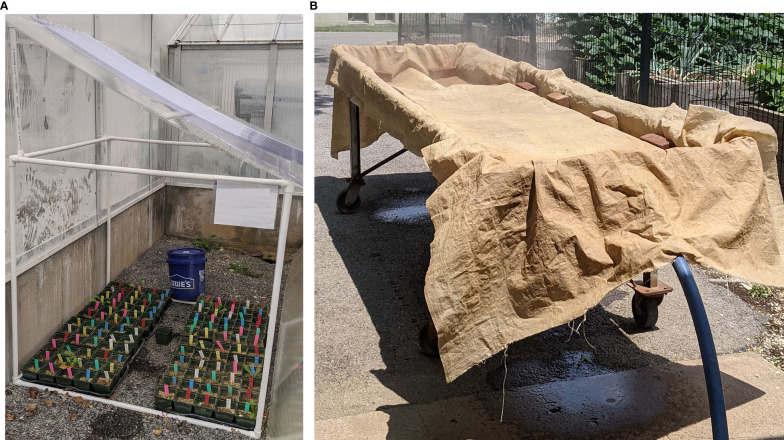
**(A)**. The soil steaming experiment was carried out at the Pennsylvania State University College of Agricultural Science’s greenhouses over approximately 14 months. All treatment replicates were kept under an outdoor ramada constructed of PVC piping and polycarbonate roofing to prevent rain splash (cold frame). **(B)** Pots were filled with soil and were steamed at 82°C (180°F) within a metal steaming table for 1 hour. The steam hose was attached to a central steam line in the table, the items to be steamed were placed along the central steam line and a heat-resistant tarp was placed over the items and weighed down with bricks.

An experiment was conducted in the cold frame of a greenhouse (outdoor area) to understand how the fungal microbiome rebuilds in potted soil after steaming and to investigate the effects of the early introduction of a beneficial (*T. harzianum)* and the soilborne pathogen (*F. solani*). Field soil was collected from the Pennsylvania State University, Plant Pathology Farm. The top 15 to 30 cm of soil was harvested using standard shovels from the edge of a fallow field in early July 2021. The soil was collected from a single 3 m square patch of soil. Field soil was thoroughly mixed with sand and perlite at the Pennsylvania State University (PSU) College of Agriculture greenhouses for a final ratio of 3:1:1 respectively. The prepared soil mixture was dispensed into 120 four-inch (10.16 cm) pots. These pots were placed three pots by five pots in to eight shallow trays (51.44 x 31.12 cm). The trays were distributed evenly along the central steam line in a metal steaming table. The steaming table was covered with a tarp and the soil was steamed at a maximum temperature of 82°C (180°F) and was held at this temperature for one hour (**Figure 1**) (Fennimore and Goodhue, 2016; Arancibia, 2020).

**Figure 2:**
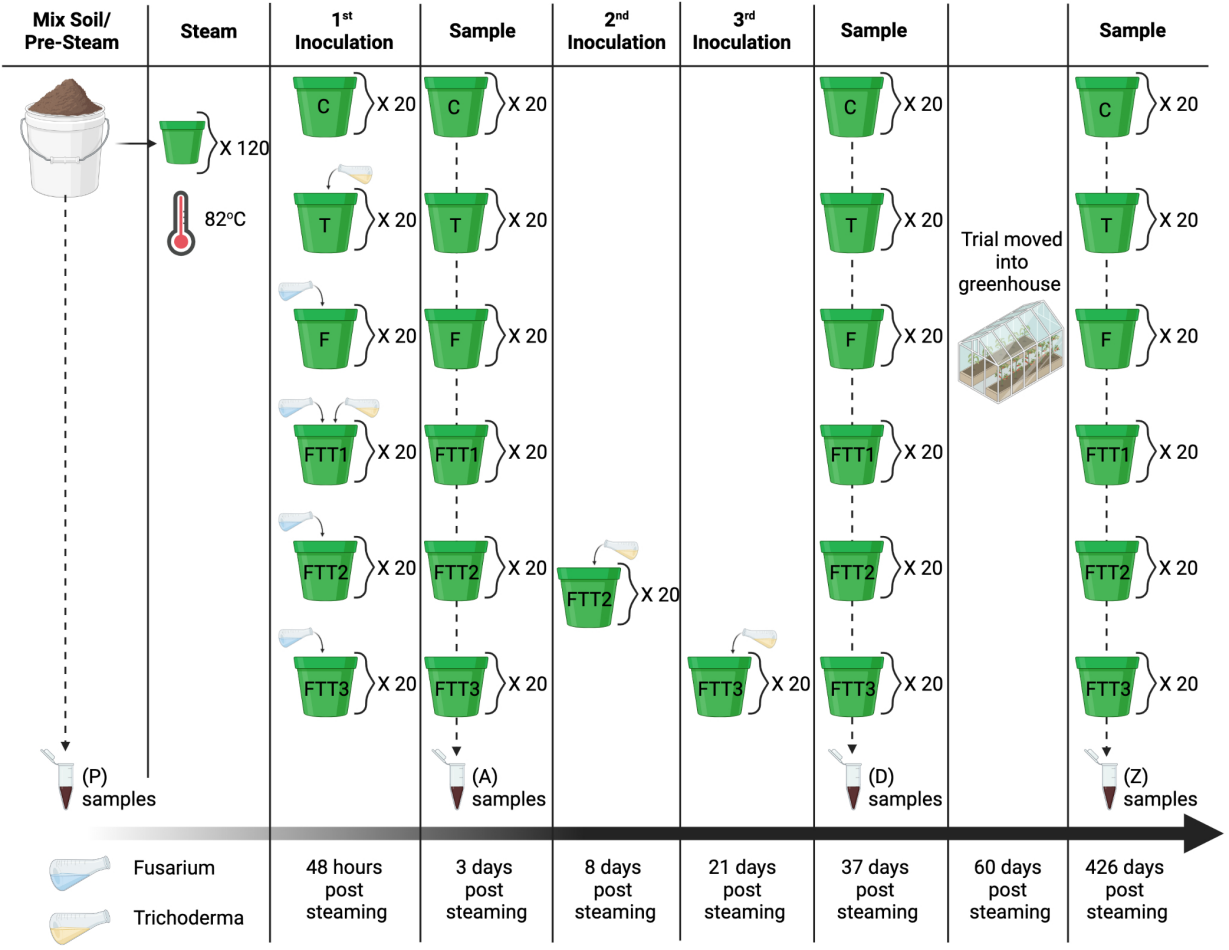
Experimental design; field soil was mixed with perlite and sand and sampled at the pre-steam time point (P). The soil was distributed into 120 pots and steamed to 82°C (180°F) for one hour. The first round of inoculations was applied 48 hours post steaming. The treatments were the control (C), Trichoderma (T), Fusarium (F), Fusarium Trichoderma Time 1 (FTT1), Fusarium Trichoderma Time 2 (FTT2), and Fusarium Trichoderma Time 3 (FTT3). The first samples were taken 3 days post steaming. *Trichoderma harzianum* was added to the FTT2 replicates eight days post-steaming and to the FTT3 replicates 21 days post-steaming. The second set of samples (D) were taken 37 days post steaming. At the end of the summer the experiment was moved into a greenhouse. The last set samples (Z) were taken 426 days post steaming.

The pots containing steamed soil were left to cool for 24-hours under the steaming tarp (**Figure 1**). The pots were then moved to an outdoor gravel cold frame structure behind the greenhouses (**Figure 1**). The experimental trial was initially kept outside as aerial spore dispersal is one way that new microbes can colonize the soil after the steaming disturbance and we assumed that the airborne fungal spore density is naturally greater outside than inside (Warcup, 1955; Cho et al., 2018; Martinez-Bracero et al., 2022). The pots were partially protected from rain by a 1.2 x 1.2 meter cube ramada, with a 0.61-meter roof, constructed of PVC piping with a polycarbonate roof to prevent cross contamination from rain splash (**Figure 1**). Treatments were first applied 24-hours after being placed in the cold frame (48 hours post steaming) (**Figure 2**). The experiment ran in the cold frame over the summer of 2021 (July–September). Over the course of the experiment each pot was watered twice a week with 20 ml of tap water. In September 2021 the pots were moved into a greenhouse where they were maintained for an additional year (Sept. 2022).

### Soilborne pathogen (*Fusarium solani*) & biocontrol (*Trichoderma harzianum*) treatments

There were six treatments with 20 replicates each. The control (C) treatment was steamed soil with no further amendments. The remaining treatments were: steamed soil with *Fusarium solani* (F), steamed soil with *Trichoderma harzianum* (T), steamed soil with *F. solani* and *T. harzianum* at time one (FTT1), steamed soil with *F. solani* and *T. harzianum* at time two (FTT2), and steamed soil with *F. solani* and *T. harzianum* at time three (FTT3) (**Figure 2**).

All treatments, excluding the control, were inoculated with *F. solani* 48-hours post steaming (48hps) (**Figure 2**). The *F. solani* was isolated from a Pennsylvania soybean field and pathogenicity was confirmed with an *in-vitro* seedling assay (personal communication, Dilooshi Weerasooriya, Jun 9, 2021). Fifteen Potato Dextrose Agar (PDA) culture plates of *F. solani* were flooded with 5 ml of sterile 0.01% Tween20 deionized (DI) water and scrapped with a glass rod to harvest microconidia. The concentration of microconidia was calculated using a hemocytometer and diluted with sterile DI water to a final concentration of 2e6. The microconidia suspension was applied at a rate of 1 ml per 100 g of soil. The pots averaged 329 g of soil, and the *F. solani* inoculum was applied at 3.5 ml per 4-inch pot (Leslie and Summerell, 2008).

The commercially available product RootShield® was used for the *Trichoderma* spp. treatments. The active ingredient in RootShield® is *T. harzianum*. RootShield® is commonly used in the nursery industry and its efficacy against *Fusarium* spp. in greenhouse production has been studied (Lamboy and Call, 2001; Rose et al., 2003; Roberti et al., 2012). RootShield® was applied at the recommended concentration for nursery pots (3–4 oz of powdered RootShield® per 100 gallons of water applied at 4–8 oz per 3–6inch pot). This resulted in 0.264 grams of powdered RootShield® per liter of DI water applied at 120 ml per 4-inch pot. Treatment FTT1 had *T. harzianum* applied in conjunction with *F. solani*. Treatment FTT2 had *T. harzianum* applied two weeks after *F. solani*, and treatment FTT3 had *T. harzianum* applied three weeks after *F. solani* (**Figure 2** *)*. This was a completely randomized designed experiment conducted in plant pots. To decrease the influence of weather and wind patterns, the pots were randomly moved within the cold frame 3 separate times over the course of the experiment.

### Sample collection

The day that the soil was mixed and steamed, a pre-steam soil sample was collected prior to steaming the pots. After steaming, samples were collected 24 hours after each treatment was applied, two weeks after the last treatment was applied, and around a full year after the initial steaming. The number of samples collected for DNA sequencing to identify microbial communities was limited by funding, resulting in the selection of 3 of the sampling time points; 3 days post steaming, 37 days post steaming, and 426 days post steaming. Soil samples were collected using sterile plastic transfer tubes and stored in small plastic bags. The tubes were washed and autoclaved before each sampling. The 4-inch pots were gridded three by three, and at each sampling two quadrants were randomly selected for sampling. The transfer tubes were used to collect samples that transected the entire soil column of each pot. Samples were stored on ice and then transferred to long term storage in a −20°C freezer. These samples were stored for downstream DNA extraction, sequencing and analysis to identify changes in the soil microbiome over time (**Figure 2**).

### Soil DNA extraction, quantification, & sequencing

Frozen soil samples were thawed and mixed to fully homogenize each sample. A subset of each sample was weighed out to be extracted, 250 to 300 grams of soil per extraction. Extractions were done using the QIAGEN DNeasy® PowerSoil Pro Kit (QIAGEN, Carlsbad, CA, USA) to the manufacturers specification (Lear et al., 2018; Pearman et al., 2020). The DNA yield in our samples (ng/μL) DNA were calculated for all 161 extracted samples using a Qubit for broad range dsDNA. The DNA concentration data were analyzed using a one-way ANOVA and *post-hoc* Tukey analysis. We used metabarcoding (amplicon-based sequencing) to determine the composition and relative abundance of the fungal microbiome in our soil samples. The ITS PCR primers (Forward: 5’CTT GGT CAT TTA GAG GAA GTA A-3’, Reverse 5’-GCT GCG TTC TTC ATC GAT GC-3’) (Gardes and Bruns, 1993) were used for PCR amplification with HotStarTaq Plus Master Mix Kit (Qiagen, USA) under the following conditions: 95°C for 5 minutes, 30 cycles of 95°C for 30 seconds, 53°C for 40 seconds and 72°C for 1 minute, and a final elongation step of 72°C for 10 minutes. PCR products were checked using a 2% agarose gel. Samples were multiplexed using unique dual indices and pooled together in equal proportions based on their molecular weight and DNA concertation. Samples were purified with calibrated Ampure XP beads prior to preparing an Illumina DNA library. Sequencing was performed at MR DNA (Shallowater, TX, USA) on a MiSeq Illumina Platform following the manufacturer’s guidelines.

### Metabarcoding bioinformatics pipeline and statistical analysis

The MR DNA bioinformatics pipeline was used to generate Amplicon Sequence Variants (ASV) and taxonomy tables which were further processed using the phyloseq package in R (MR DNA, Shallowater, TX, USA). The pipeline sequences were first joined, then sequences with less than 150bp and/or ambiguous base calls were removed. The sequences were then quality filtered using a maximum expected error threshold of 1.0 and dereplicated and denoised to provide a final ASV. Taxonomy was assigned using BLASTn against a curated database derived from NCBI (www.ncbi.nih.gov). Once taxonomy was assigned using the pipeline, we employed the phyloseq package in R to filter, sort, normalize, agglomerate, and graphically and statically analyze the genera counts in order to determine the effects of our treatments on the composition of the soil fungal microbiome (McMurdie and Holmes, 2013). To avoid spurious taxonomic designations, genera with a prevalence of less than 0.0000005 were filtered out, removing 51 genera. For abundance counts the top 20 genera were used for graphical analysis and the low abundance genera were grouped into a single category. The alpha diversity was graphically analyzed using the estimate_richness function for the observed index, Shannon index and Inverse Simpson index. We ordinated the genera data using Bray-Curtis distances in a principal coordinates analysis (PCoA). Statistical differences were analyzed by pairwise PERMANOVAs using the pairwiseAdonis package in R (Martinez, 2020). Three different sets of PERMANOVAs were conducted to make specific comparisons. The first PERMANOVA compared the effect of treatment, combining the treatment data from each timepoint. The second set of PERMANOVAs compared each treatment across time by creating one PERMANOVA for each treatment. Lastly, a set of three PERMANOVAs was run, one for each time point, to compare the treatments at each time point independently. Finally, in order to understand what the broad ecological functions were of the fungal microbiomes found in our experiment, we employed the FUNGuild tool (Nguyen et al., 2016). FUNGuild is an open annotation community resource tool based in python that is used to parse fungal ASVs by ecological guild (e.g., plant pathogen, saprotroph, beneficial) and draws from guild assignments from the literature. This pipeline functions independently of sequencing platforms and analysis pipelines (Nguyen et al., 2016). This database is accessible at https://github.com/UMNFuN/FUNGuild.

### Soybean germination experiment

In order to assess plant response to the altered fungal microbiomes, a pilot soybean germination experiment was conducted. A single soy seed was planted in each pot after the final microbiome sampling (1 year post-steaming). Susceptible seeds of the SC9277R variety were used. A subset of seeds were destructively sampled 18-hours post-planting for further analysis in a different study. The remaining 12 replicates were visually observed until successful germination. Data was not collected post germination as the pots were too small to support plant growth. The percent germination rate and days to germination were both compared via one way ANOVA.

## Results

### Soil steaming treatments over time

This experiment was conducted over a 14-month period. Specifically, the samples were collected at 4 time points: pre-steam (after the soil was mixed, immediately before steaming), 3 days post steaming, 37 days post steaming, and 426 days post steaming (hereafter, dps) (**Figure 2**). We compared the taxonomic similarity between fungal soil communities using PERMANOVA pairwise comparisons.

The first PERMANOVA compared *the effect of treatment on fungal community composition*, combining the treatment data from each timepoint. This PERMANOVA showed that almost all of the experimental treatments (including the mock fungal community and the negative controls) were significantly different from one another in taxonomy. However, treatments that were not significantly different from one another were those that contained the pathogen *F. solani*: Fusarium (F) vs. Fusarium*Trichoderma Time 3 (FTT3), Fusarium*Trichoderma Time 1 (FTT1) vs Fusarium*Trichoderma Time 2 (FTT2), FTT1 vs FTT3, FTT2 vs FTT3.

The second set of PERMANOVAs were conducted to *compare individual treatments across time points*, including the pre-steam time point. These PERMANOVAs revealed that regardless of treatment, all steamed soil was significantly different than the pre-steam samples at every time point (0.001 ≤p≥ 0.036).

The third set of PERMANOVAs were employed to *run pairwise comparisons between each treatment to quantify if fungal community composition was different at each time point during soil recovery* (3dps, 37dps, and 426dps). At all three time points post-steaming, all pairwise comparisons were significantly different from each other except the treatments where *F. solani* was added. At 3dps F was not significantly different than FTT2 or FTT3, and FTT2 and FTT3 were not significantly different. At 37dps the F was not significantly different than FTT1 and FTT1, FTT2, and FTT3; these in turn were not significantly different from each other. By 426dps none of the fungal communities containing *F. solani* were significantly different from each other. At 426dps, fungal communities in F were significantly different than the control (steamed-only) (p = 0.001) while fungal communities found in the Trichoderma-only (T) treatment were only moderately different from the control (p = 0.024) (**Table 1**).

**Table 1:** A pairwise PERMANOVA was run between each treatment to quantify if fungal community composition was different at each time point during soil recovery (3dps, 37dps, and 426dps).

Pairwise Comparisons	3 dps (A)		37 dps (D)		426 dps (Z)	
	R sq.	p-value	R sq.	p-value	R sq.	p-value
C vs F	0.519	0.007*	0.362	0.020.	0.319	0.001*
C vs T	0.470	0.007*	0.180	0.029.	0.153	0.024.
C vs FTT1	0.653	0.001*	0.329	0.013.	0.309	0.001*
C vs FTT2	0.670	0.014.	0.322	0.012.	0.333	0.001*
C vs FTT3	0.670	0.008*	0.297	0.002*	0.336	0.001*
F vs T	0.660	0.013.	0.499	0.003*	0.279	0.001*
F vs FTT1	0.380	0.004*	0.163	0.087	0.074	0.292
F vs FTT2	0.231	0.066	0.365	0.009*	0.095	0.102
F vs FTT3	0.239	0.065	0.211	0.055.	0.067	0.431
T vs FTT1	0.592	0.008*	0.321	0.007*	0.259	0.001*
T vs FTT2	0.812	0.009*	0.256	0.006*	0.216	0.002*
T vs FTT3	0.810	0.014.	0.251	0.025.	0.289	0.002*
FTT1 vs FTT2	0.692	0.008*	0.153	0.108	0.046	0.830
FTT1 vs FTT3	0.684	0.014.	0.085	0.781	0.034	0.948
FTT2 vs FTT3	0.044	0.903	0.088	0.730	0.070	0.389

The six soil treatments were; control (C), Fusarium (F), Trichoderma (T), Fusarium Trichoderma Time 1 (FTT1), Fusarium Trichoderma Time 2 (FTT2), and Fusarium Trichoderma Time 3 (FTT3). Significance was shown by (*) and moderate significance by (.).

**Figure 3:**
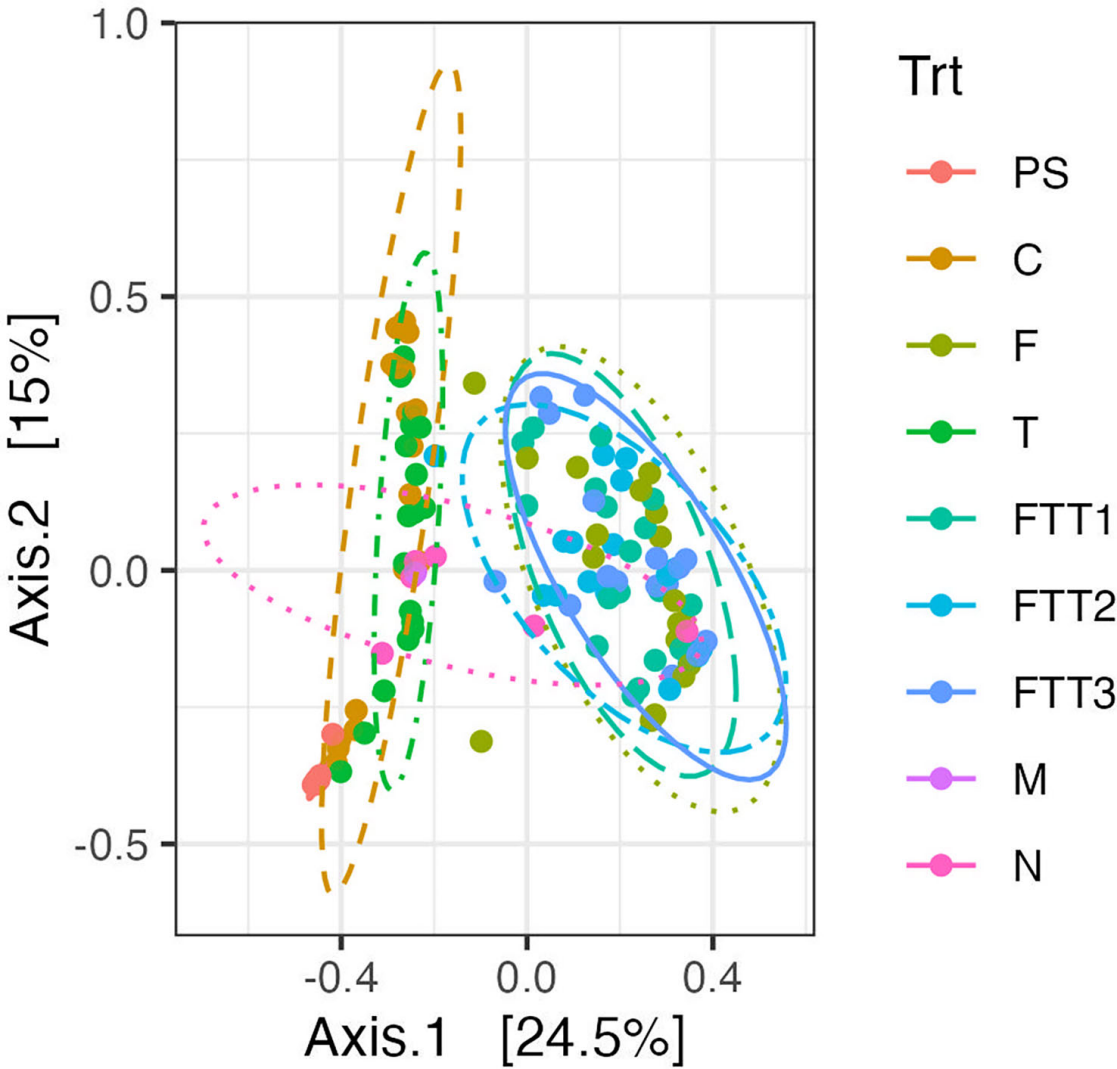
A principal coordinate analysis (PCoA) using the Bray-Curtis dissimilarity index was constructed for the six soil treatments; control (C), Fusarium (F), Trichoderma (T), Fusarium Trichoderma Time 1 (FTT1), Fusarium Trichoderma Time 2 (FTT2), and Fusarium Trichoderma Time 3 (FTT3). The pre-steamed soil (PS), the mock community (M), and the negative controls (N), are included in the plot. 95% confidence interval ellipses were drawn. There were two distinct groupings. The first: C and T the second: F, FTT1, FTT2, and FTT3.

A principal coordinate analysis plot (PCoA) was used to visualize whether the fungal microbiomes found within each of the treatments were similar to one another. We added 95% confidence intervals to our ordination as ellipses. The first PERMANOVA revealed no differences between the FTT1, FTT2, and FTT3 treatments. In the PCoA plot the three ellipses for these treatments were visually stacked upon each other, indicating that these communities were more similar in composition to each other than other microbial communities (**Figure 3**). The F treatment was also grouped within these three treatments in the PCoA (**Figure 3**). The F treatment was not significantly different than FTT3, but was from FTT1 (p=0.016) and FTT2 (p=0.035). However, these p-values maybe be skewed because two F replicate samples were clearly outliers. Without these outliers the F treatment may be more similar to FTT1, FTT2, and FTT3. The pre-steam replicates are grouped the tightest together, with the narrowest 95% confidence interval (**Figure 3**). Although statistically different, the control treatment (steam only) and T treatments grouped together and were clearly distinct from all treatments containing *F. solani* (**Figure 3**).

### Fungal community composition, diversity, & ecological function

We created relative abundance bar graphs (rainbow plots) of the top 20 most abundant genera to visualize which fungal genera were present in our samples. This analysis was to determine the changes in the fungal microbiome over time and across treatments. The public database FUNGuild was used to assign the genera to ecological guilds; we conducted this analysis in order to better determine changes in broad ecological roles and functions (e.g., pathogens, saprotrophs, beneficials) of the soil fungal microbiome (Nguyen et al., 2016) (**Supplemental 1**).

**Figure 4:**
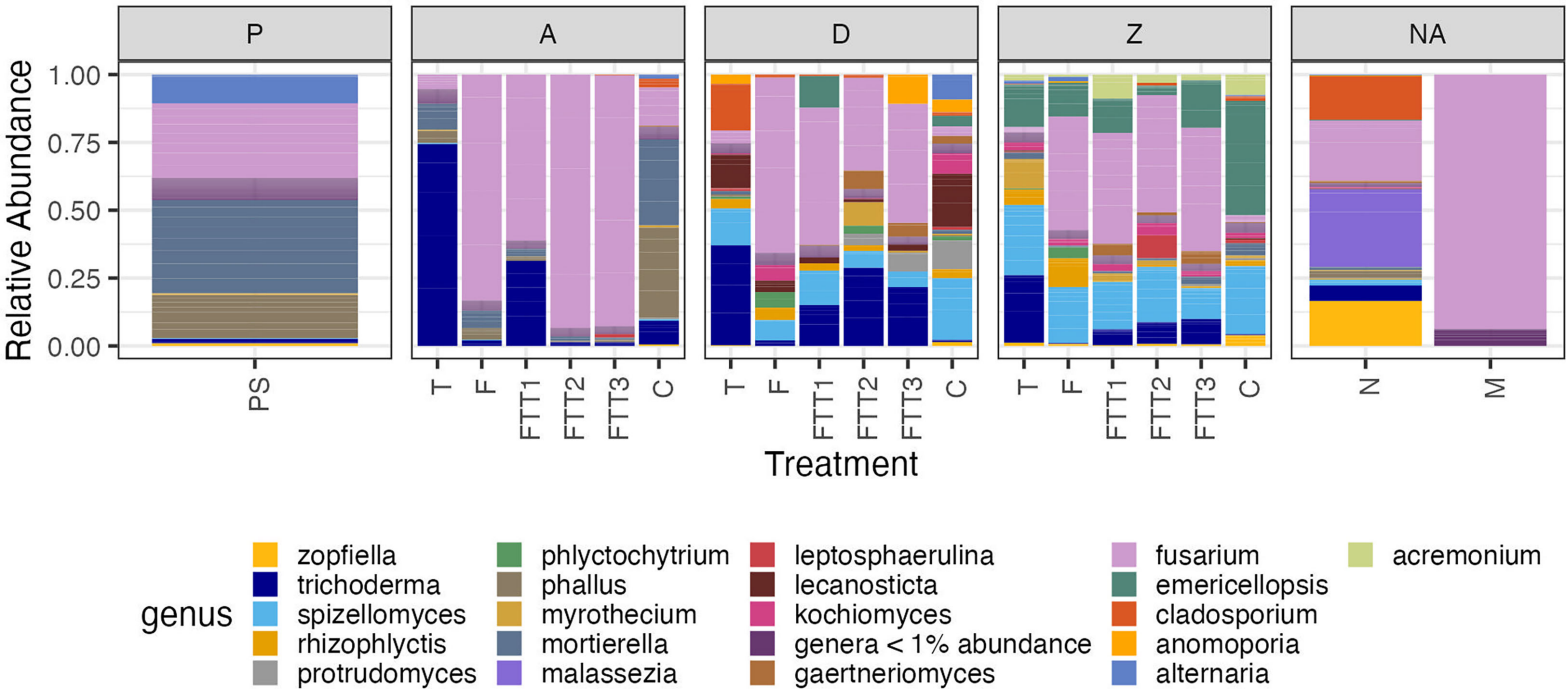
The top 20 most abundant genera for the soil steaming, pathogen, and beneficial soil addition experiment. Treatment is on the x-axis, and relative read abundance on the y-axis. The treatments were: pre-steam (PS), Trichoderma (T), Fusarium (F), Fusarium Trichoderma Time 1 (FTT1), Fusarium Trichoderma Time 2 (FTT2), Fusarium Trichoderma Time 3 (FTT3), control (C), negative control (C), mock community (M). The plot was faceted by the sampling time points; pre-steam (PS), 3 days post steaming (A), 37 days post steaming (D), 426 days post steaming (Z), and time not applicable (NA).

Before the soil was steamed there were four dominant genera (*Mortierella* sp., *Fusarium* sp., *Alternaria* sp., and *Phallus* sp.) and four genera that were naturally present at low abundancies (<5%, *Trichoderma* spp., *Zopfiella* spp., *Spizellomyces* spp., and *Mycrothecium* spp.) (**Figure 4**). At time 3dps the four dominant pre-steam plant pathogen genera were present in the untreated control: *Alternaria* spp. was no longer found to be a dominant genus and *Cladosporium* spp. appeared. The FUNGuild analysis determined that the fungi present in the pre-steam soil and the control at 3dps were categorized as saprotrophs, endophytes, plant pathogens, fungal parasites, and other guilds that comprised a combination of these ecological roles (**Supplemental 1**). A pairwise PERMANOVA returned an adjusted p-value of (0.001) indicating a significant difference between pre-steamed soil and the control at 3dps. The Shannon diversity and inverted Simpson indices were both higher in the pre-steamed soil than the control at 3dps (**Supplemental 2**). The 95% confidence interval did overlap in the PCoA (**Figure 3**).

At 3dps the Trichoderma, Fusarium, and combination treatments all had relative abundances that were dominated by a single fungus. The Fusarium, FTT2, and FTT3 treatments all had *Fusarium* at >75% relative abundance. The Trichoderma treatment had *Trichoderma* spp. at >75% relative abundance. The FTT1 treatment had *Fusarium* spp. at >50% relative abundance and *Trichoderma* spp. >25% relative abundance (**Figure 4**). The guilds present in these four treatments at 3dps were the same guilds present in the control and pre-steam treatments (**Supplemental 1**). The Trichoderma treatment was significantly different than the pre-steamed soil (p=0.002), as was the Fusarium treatment (p=0.004), FTT1 (p=0.005), FTT2 (p=0.007), and FTT3 (p=0.002) (**Table 1**). At 3dps the Fusarium, FTT2, and FTT3 treatments were not significantly different from each other, but all other treatments were (0.007 < p > 0.014) (**Table 1**). The inverse Simpson index was low for all five treatments. The Shannon index was lower for all five than for the pre-steam and control treatments but it was slightly higher for Trichoderma (**Supplemental 2**).

At 37dps, in all treatments where *F. solani* was applied, we found members of the *Fusarium* genus to have the highest relative abundance of any genus in the fungal microbiome. All treatments where both *T. harzianum* and *F. solani* were applied showed that *Trichoderma* spp. exhibited the second highest relative abundance (**Figure 4**). At 37dps all treatments with *Fusarium* were the most similar. The Fusarium treatment was not significantly different than FTT1 or FTT3 (**Table 1**). All other pairwise comparison resulted in significant differences (0.002 ≤ p ≥ 0.029) (**Table 1**). When examining the functional guild data for the Fusarium treatment, plant pathogens constituted nearly 75% of the relative abundance of all taxa within the fungal microbiome (**Supplemental 1**). The remaining microbiome comprised almost solely saprotrophs and possibly other plant pathogens. Endophytes, ectomycorrhiza, and arbuscular mycorrhiza were present at low relative abundancies <5% (**Supplemental 1**). The only notable difference between the Fusarium treatment and the FTT1, FTT2, and FTT3 was the relative abundance of endophytes, which was attributed to the *Trichoderma* spp. The control (steam-only) had saprotrophs, plant pathogens, and “possible” plant pathogens at >75% relative abundance. The Trichoderma treatment possessed endophytes at >25% relative abundance (which was the *Trichoderma* spp.) and the relative abundance of saprotrophs, plant pathogens, and possible plant pathogens, dropped <75%. There was an increase in the relative abundance of arbuscular mycorrhiza compared to the control (**Supplemental 1**).

By 426dps all soil treatments where *F. solani* was applied had roughly 40% relative abundance of *F. solani* (**Figure 4**). When applied on its own to the soil, *F. solani* held a higher relative abundance than *T. harzianum* applied on its own. At 426dps, the Trichoderma treatment had a relative abundance of *Trichoderma* spp. of 25% (**Figure 4**). There was no significant difference between any of the treatments that contained *F. solani*, while all other pairwise comparison resulted in significant differences (0.001 < p > 0.002) (**Table 1**). The control treatment and Trichoderma treatment were significantly different but at a much higher p-value than all other comparisons (p=0.024) (**Table 1**). At 426dps the diversity indices were evenly distributed across soil treatments (**Supplemental 2**). The mean Shannon index was roughly even for the Trichoderma treatment and the control (**Supplemental 2**). The inverted Simpson index was low for all treatments at 426dps, but it was just slightly higher for both the Trichoderma and control treatments (**Supplemental 2**). Across all treatments there was a rise in saprotrophs and plant pathogens as the soil recovered from steaming (**Supplemental 1**).

Soybean seeds were planted in the soil toward the end of the experiment at 480dps to measure both germination rate and time to germination for each treatment. Percent germination was equal across all treatments. A one-way ANOVA was conducted to access if there was a difference in days to germination from planting. Although not statistically significant, we observed that within the Fusarium treatment, the soybeans took longer to germinate (median time was 2 days longer) compared to the rest of the treatments (**Supplemental 3**).

### Quantifying DNA yield

There was a significant difference in DNA yield (ng/μl) between the sampling time points (p=2e-16). However, there were no significant differences found within treatments or treatment interactions between the sampling time points. The Tukey Test analysis showed significant difference between each sampling timepoint (**Supplemental 4**). Specifically, the pre-steamed soil had an average DNA yield of 80.16ng/μl, soil sampled 426dps had an average DNA yield of 21.74ng/μl, soil sampled 37dps had an average DNA abundance of 10.79ng/μl, and soil sampled 3dps had an average DNA abundance of 3.01ng/μl (**Supplemental 4**).

## Discussion

Today, a flurry of research papers have been published that use culture independent methods to quantify the recovery of the soil fungal communities after an environmental disturbance (Bell et al., 2013; Bowd et al., 2022; Wydro, 2022). Many of these studies incorporate high throughput sequencing to obtain a holistic picture of the taxa present and their gene and metabolic functions (Stefani et al., 2015; Crandall et al., 2022). We know soil microbiomes are sensitive to disturbance events and that their recovery is impacted by abiotic and biotic factors (Shade et al., 2012; Thakur and Geisen, 2019). However, there is currently a poor understanding of how microbial interactions shape soil microbiome dynamics and recent studies view microbes as regulators of the soil microbiome structure, particularly for the competition for resources (Shade et al., 2012; Bahram et al., 2018; Thakur and Geisen, 2019). Manipulative experiments are rarely conducted where microbes are grown *in-vivo* within soil to investigate the ecological roles of microbes such as a plant pathogenic fungus, a beneficial fungus, and their correlative interaction between the soil microbiome. In this paper, we experimentally quantified how the soil microbiome changes after adding the plant pathogen *F. solani* and its microbial antagonist, the beneficial microbe *T. harzianum*. As the soil microbiome directly affects plant health by facilitating or hindering seed germination, we also tested how steaming and our pathogen-beneficial soil additions would affect soybean germination rate and timing. We discuss 3 major findings from our experiment and general conclusions.

First, early introduction of the pathogen *F. solani* post-steaming decreased the diversity and relative abundance of other fungi in the soil fungal microbiome. We originally hypothesized that the addition of *F. solani* would substantially decrease the soil fungal community composition and diversity when compared to pre-steamed soil because it is known to grow quickly and aggressively in the soil once present. *Fusarium solani* is also known to intensify resource competition between microbes in the soil (Celar, 2003; Wei et al., 2019). After adding *F. solani* post-steaming we observed a phenomenon similar to dysbiosis in the human gut: when one to a few dominant, aggressive pathogens rapidly infect and outcompete other biota after broad-spectrum antibiotics eliminate gut communities (Fröhlich et al., 2016). In our system, we found that when inoculated into post-steamed soil, *F. solani* immediately, significantly, and persistently changed the fungal microbiome (**Figures 3**, **4**; **Table 1**).

The most notable finding was the increase in *F. solani*’s dominance in the soil when compared to other microbes. At 3 days post steaming (24 hours post *F. solani* inoculation), soil that was inoculated with *F. solani* had high relative abundances of *Fusarium* spp. (**Figure 4**). It is important to note that not all “*Fusarium”* were taxonomically assigned as *F. solani* using the metabarcoding approach. This could be explained by *Fusarium*’s complex genetics and the lack of records for unidentified fungi that are not within the reference library to assign names to our fungal taxonomy. Regardless of the species, the high abundance of the genus *Fusarium* spp. that naturally colonized and was experimentally added was clear (**Figure 4**). The relative abundance of *Fusarium* spp. in all treatments where *F. solani* was added did diminish over time but was sustained at a steady relative abundance of 40% compared to the rest of the fungal microbiome (**Figure 4**). The fungal microbiomes of all treatments with added *F. solani* were significantly different from the Trichoderma-only treatment and control (steam-only) treatment at every time point and the pre-steamed soil. By the last timepoint (426dps) none of the four treatments with *F. solani* were significantly different from each other (**Table 1**). By 37dps the three Trichoderma Fusarium co-treatments were not significantly different from each other. At 37dps only the Fusarium Trichoderma Time 2 treatment had communities that were significantly different than the Fusarium-only treatment. In the context of the microbiome’s recovery post-steaming, this result suggests that the soil microbiomes within *F. solani* treatments reached a new steady state by 37dps. This 1–2 month time for microbial community stabilization is corroborated by previous research (Crandall et al., 2022). At this time point the treatments containing *F. solani* were almost all statistically non-different (**Table 1**) and their compositions looked similar from 37dps and 426dps (**Figure 4**). The similarities found among all *F. solani* treatments, regardless of *T. harzianum*, was not what we expected to see, which brought into question the magnitude of the impact on *F. solani* suppression of adding *T. harzianum*.

Second, *Trichoderma harzianum* did not strongly mitigate the effect of *F. solani* on the soil fungal microbiome*. Trichoderma* spp. have been grown and sold as promising biocontrol agents to manage a whole suite of soilborne pathogens (Lamboy and Call, 2001; Rose et al., 2003; Roberti et al., 2012; Sood et al., 2020). Certain *Trichoderma* spp. either alone or in combination, can increase plant health by facilitating the formation of mycorrhizal associations and can combat pathogens by out competing them for nutrients, space, and by acting as a mycoparasite (Daniel and Filho, 2007; Pimentel et al., 2020; Sood et al., 2020). *Trichoderma harzianum* has been shown to specifically aid in controlling *F. solani* in a number of cropping systems by increasing plant health, competing with *F. solani* for resources, and by acting as a mycoparasite (Steindorff et al., 2012; Ben Amira et al., 2017; Erazo et al., 2021). These findings on mycoparasitism led us to hypothesize that the application of *T. harzianum* after *F. solani* would result in a significant reduction in the relative abundance of *F. solani*, thereby reducing its impact on the microbiome. This result was not found. At each time point the Trichoderma-only treatment was significantly different from each of the Fusarium Trichoderma co-treatments, and by 426dps there was no significant difference between the Fusarium-only treatment and the co-treatments (**Table 1**). As mentioned before, by 37dps none of the co-treatments were statistically different from one another, indicating that the timing of *T. harzianum* application post *F. solani* application had no effect on their interaction (**Table 1**). When taken together, these results suggest that *T. harzianum* did not strongly mitigate the effects of *F. solani* on the fungal community. Although *T. harzianum* did not seem to decrease the relative abundance of *F. solani*, it did have its own significant impact when compared to the steam-only control.

Third, similar to *F. solani*, the beneficial microbe *T. harzianum* decreased the diversity of the soil fungal microbiome compared to the steam-only control, however, the effect was dampened compared to the effect of *F. solani*. Inoculating *T. harzianum* at 48hps resulted in a high relative abundance of *Trichoderma* at 3dps (**Figure 4**). Also similar to the *F. solani* treatments, not all *Trichoderma* that naturally colonized the soil were assigned as *T. harzianum* when conducting our taxonomic analysis. Unlike the levels of *Fusarium* spp., *Trichoderma* spp. did not maintain their high relative abundance across the proceeding timepoints. When compared to the control (steam-only), the Trichoderma-only treatment was significantly different at all timepoints. This difference was the least significant at 426dps (p=0.024). Although the PERMANOVA determined the Trichoderma-only treatment to be significantly different than the control, the PCoA showed clear overlap of the Trichoderma-only and control (steam-only) treatments (**Figure 3**), suggesting very similar communities. This overlap created a distinct group separate from the treatments with *F. solani* (**Figure 3**).

Another important microbiome parameter to consider is fungal diversity. The Shannon Diversity Index was highest for the control at all time points post steaming and the second highest for the Trichoderma-only treatment (**Supplemental 1**). This pattern was also seen in the inverse Simpson Diversity Index (**Supplemental 1**). Diversity does not necessarily mean a healthier microbiome, but a major concern for a highly competitive biocontrol agent such as *T. harizanum* is that it will out compete other microbes in the soil for resources and/or space with no discretion for pathogen or beneficial. We found that this was not the case with *T. harizanum* as it rapidly colonized during the start of the experiment, but its relative abundance fell over time. The lower abundance may have allowed for a more diverse fungal microbiome to develop, a microbiome that was more similar to the untreated control (**Figure 3**).

Despite the similarities and differences among the post-steamed treatments, we have to consider the pre-steamed soil. All treatments at all time points were significantly different from the pre-steamed soil. This result suggests after the steaming disturbance, the trajectories of the fungal soil microbiomes diverged from the original soil fungal microbiome. The diversity indices (Shannon and inverse Simpson) were notably higher for the pre-steamed soil (**Supplemental 2**). The DNA quantification data showed that in the pre-steam soil we were able to extract 80.16ng/ml, which is roughly four times more than at 426dps and 26 times more than at 3dps (**Supplemental 4**). Even after a year of fungal community assembly within each treatment, the amount of DNA in the soil was nowhere close to where it had been pre-steaming. This indicated that steaming did denature the DNA and we were not sampling and counting dead organisms. The top 20 genera present in the pre-steam soil greatly resembled those present in the control treatment at 3dps (**Figure 4**). The control treatment did significantly change at the later 2 time points. We surmise that recruitment of soil fungal microbiome was from aerial spores, rain splash, and possibly invertebrate movement because the experiment was in an outdoor cold frame setting. The setting was intentional, as we wanted to mimic the colonization of microbes in soil in outdoor environments. This might explain the similarity of the most abundant fungi in the control at 3dps to the pre-steamed soil. A separate study that examines aerial spores sampled from soil transects is necessary to understand which spores land on the soil surface and the extent of mycelial growth within the soil column. In the same vein, it would be beneficial to conduct a future experiment where pre-steamed soil is included as a treatment across all time points. This experiment was designed to investigate the rebuilding of the soil-microbiome post steaming, so the experiment was designed for the post-steaming time points with the intention of comparisons being made between post-steaming treatments. In hindsight, the benefit of a pre-steam treatment became clear. We cannot conclude if the fungal microbiome of the pre-steamed soil would have changed in similar ways to the control (steam-only) across the experiment. When looking at the PCoA where shape indicated sampling time it was clear that overtime all of the samples were moving in the same direction across the plains (**Supplemental 5**). We do not know if the pre-steam samples would have moved in the same way.

Diversity and abundance do not necessarily correlate to the health and function of the microbiome and soil (Shade et al., 2012). In order to better describe the various ecological roles of the fungal communities and assign functional groups in our experiment, we used FUNGuild, a database that provides a coarse-grain interpretation of fungal ecological roles. The main takeaways from this analysis were that over time there was an increase in saprotrophs and plant pathogens across all treatments and the Trichoderma-only treatment possessed more endophytes than the control. The later finding may be because many *Trichoderma* spp. are categorized as endophytes by FUNGuild (**Supplemental 1**). Many of the taxon that were present across samples belonged to ubiquitous soil fungi that have many species that fall under saprobe/endophyte/pathogen, and many are opportunistic pathogens, therefore assigning them to a specific guild was simply not possible. To understand how the manipulated soil microbiomes would contribute to plant germination, we planted soybeans at the end of the steaming experiment. We found no significant difference in germination rate between treatments, however, the Fusarium-only treatment did result in notably more days to germination (**Supplemental 3**). This increase in days to germination was not seen in the Trichoderma and Fusarium co-inoculation treatments. If *F. solani* induced slowed germination, then one could postulate that *T. harzianum* was mitigating this effect. Continued plant health data was not collected as the pots were too small to sustain healthy plant growth and we did not want to confound our results with the addition of fertilizer. When examining the microbiomes composition, *T. harzianum* did not significantly mitigate the effect of *F. solani*: the PCoA did not significantly diverge from the control, but a simple germination assay shed light on a difference of function. In the co-treatments *T. harzianum* appeared to mitigate any negative effect of *F. solani* on germination. This could be a result of *T. harzianum’s* plant health promoting properties or it could be functional differences in the microbiomes that appear similar in their diversity. Determining this would require investigation with transcriptomics or proteomics.

In summary, we found that in a post-soil steaming disinfection experiment, the addition of *F. solani* was significantly correlated to the shifts in the recovery of the soil fungal communities in comparison to the untreated control (steam-only). The addition of *F. solani* reduced the diversity of the fungi that re-colonized soil and these effects were not mitigated in a significant way by the addition of *T. harzianum* in tandem with or after *F. solani*. We also found that post-steaming the addition of *T. harzianum* significantly affected the assembly and recovery of the soil fungal microbiome in comparison to the untreated control. When compared by a PCoA the fungal microbiome of the Trichoderma-only treatment still grouped with the untreated control, and the diversity of the fungi present in the Trichoderma-only treatment was not as reduced as it was by any treatment with *F. solani*. Our results suggest that soil steaming leaves the soil vulnerable to colonization and assembly of microbes that differ significantly from the pre-steamed community composition. The rapid colonization of specific fungi, in this case, an aggressive soilborne plant pathogen can impact the trajectory and recovery of the soil microbiome. *Trichoderma harzianum* is a known beneficial, and although its presence in the soil seemed to alter the developing microbiome, the microbiome with the biocontrol agent was more similar to the untreated control than treatments with *F. solani*. We cannot surmise if the fungal microbiome that developed in the untreated control increased soil fertility, but we did find a pattern of high fungal community diversity. To answer this question more deeply, more experiments are needed that focus on other soil health measurements such as the presence and shift of macro- and micronutrients that are important for plant health. This study was limited to a single pathogen and single beneficial providing a starting place to identify patterns of pathogen-beneficial driven soil microbial community dynamics. Future work could incorporate more microbial species to measure both patterns in community composition, diversity and structure and could test a gradient of pathogen and biocontrol slurry concentrations which may affect aggressiveness of *F. solani* or the efficacy of *T. harizanium*. Over time, these baseline data can inform recommendations for best management practices to growers. If certain desirable taxa are unable to recover or recruit post-steaming (e.g., nitrogen fixers, mycorrhizae), growers can decide which soil amendments to use based on their specific soil types and conditions to increase plant yield and overall health. Future research should examine the dynamics of these interactions using metagenomics to better capture the full diversity of functional genes present in each treatment at a finer taxonomic resolution. Other -omic techniques, such as metatranscriptomics, can also shed light on gene expression to answer questions about microbiome function beyond abundance and diversity estimates.

## Data availability statement

The data presented in the study are deposited in NCBI’s Sequence Read Archive (SRA), accession number PRJNA953052.

## Author contributions

EL was the postdoctoral scholar working on this project. Their main tasks and contributions were experimental design, experimental set-up, data collection, sample processing, data processing, data analysis, and writing. SC was the assistant professor; this research was conducted in their Soilborne Disease Ecology laboratory at the Pennsylvania State University. Their main tasks and contributions were collaborating on experimental design, guidance through data processing and analysis, writing and editing the manuscript, and providing funds to support the research. All authors contributed to the article and approved the submitted version.

## Supplementary material

Supplementary Figure 1 Functional guilds plotted using FUNGuild, an open annotation community resource tool, was used to group the genera found in each treatment. The relativer read abundance of the top 20 guilds are represented in the bar plot. Treatment was on the x-axis: pre-steam (PS), Trichoderma (T), Fusarium (F), Fusarium Trichoderma Time 1 (FTT1), Fusarium Trichoderma Time 2 (FTT2), Fusarium Trichoderma Time 3 (FTT3), control (C), negative control (C), and mock community (M). The plot is faceted by the sampling time points; pre-steam (PS), 3 dapys post steaming (A), 37 days post steaming (D), 426 days post steaming (Z), and time not applicable (NA).

Supplementary Figure 2 The alpha diversity in each treatment was assessed at each time point by three diversity indexes: **(A)** Shannon **(B)** Inverse Simpson **(C)** Observed. The resulting data were presented as box plots with the index value on the y-axis and the treatments on the x-axis; pre-steam (PS), Trichoderma (T), Fusarium (F), Fusarium Trichoderma Time 1 (FTT1), Fusarium Trichoderma Time 2 (FTT2), Fusarium Trichoderma Time 3 (FTT3), control (C), negative control (C), mock community (M). The plot is faceted by the sampling time points; pre-steam (PS), 3 days post steaming (A), 37 days post steaming (D), 426 days post steaming (Z), and time not applicable (NA).

Supplementary Figure 3 After the final soil sample was collected at 426 days post-steaming, a susceptible variety of soybean (SC9277R) was planted in each pot. The pots were monitored daily and the days to germination were recorded. The data are presented as a box plot with treatment on the x-axis and the days to germination on the y-axis. No significant difference was found between treatments, however, the Fusarium treatment, all soybean seeds germinated 4 days later compared to the rest of the treatments.

Supplementary Figure 4 After the DNA was extracted from each soil sample the DNA yield (ng/ml) was quantified. These data were analyzed by time point and were averaged across all treatments. The treatments were: pre-steamed soil (P), 3 days post-steam (A), 37 days post-steam (D), and 426 days post-steam (Z). Time point was found to be significant (p=2e-16). A *post-hoc*k Tukey Test confirmed that each time point to be significantly different from the others, as seen by the letters assigned.

Supplementary Figure 5 A principal coordinate analysis (PCoA) with Bray-Curtis dissimilarity index was plotted for the six soil treatments; control (C), Fusarium (F), Trichoderma (T), Fusarium Trichoderma Time 1 (FTT1), Fusarium Trichoderma Time 2 (FTT2), and Fusarium Trichoderma Time 3 (FTT3). The pre-steamed soil (PS), the mock community (M), and the negative controls (N), are also included in the ordination. 95% confidence ellipses are drawn. Shapes (e.g., circles, triangles) delineated the time point that each sample was taken at. The time points were (P) pre-steam, (A) 3 days post steaming, (D) 37 days post steaming, (Z) 426 days post steaming, and (N) no applicable time point. As the time points progressed from A to Z, the samples for each treatment moved in the positive direction on Axis.2. Samples of treatments F, FTT1, FTT2, and FTT3, also moved in the negative direction on Axis.1 from time point A to Z. This resulted in the fungal communities within each treatment converging in their taxonomic similarity over time.
