# Supplementary figures and images for: Recovery of the soil fungal microbiome after steam disinfection to manage the plant pathogen Fusarium solani
Source: Front Plant Sci. 2023 Apr 21;14:1128518. doi: 10.3389/fpls.2023.1128518 (PMC10161934; doi:10.3389/fpls.2023.1128518)

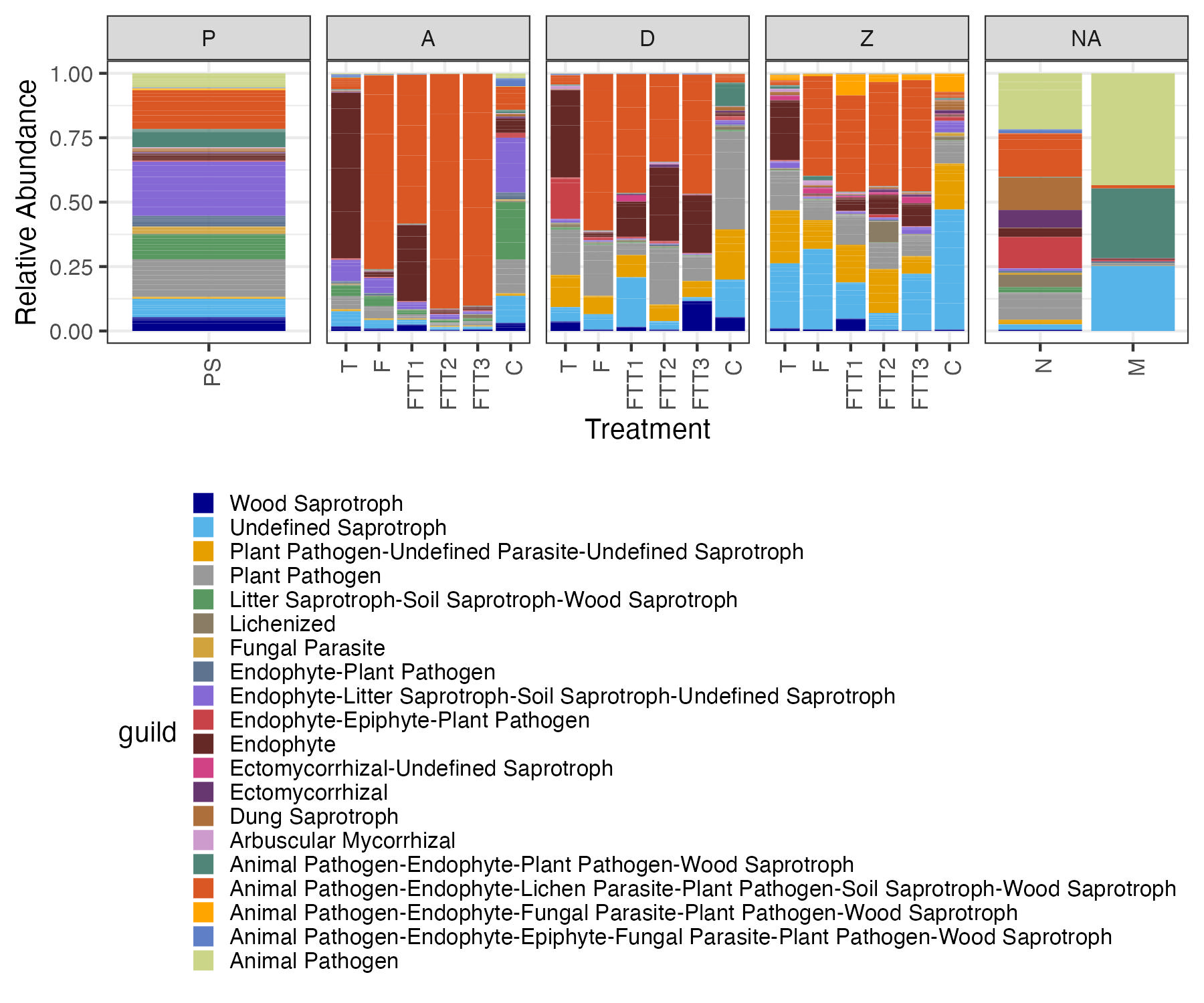

Supplement: Supplementary Figure 1 — Functional guilds plotted using FUNGuild, an open annotation community resource tool, was used to group the genera found in each treatment. The relativer read abundance of the top 20 guilds are represented in the bar plot. Treatment was on the x-axis: pre-steam (PS), Trichoderma (T), Fusarium (F), Fusarium Trichoderma Time 1 (FTT1), Fusarium Trichoderma Time 2 (FTT2), Fusarium Trichoderma Time 3 (FTT3), control (C), negative control (C), and mock community (M). The plot is faceted by the sampling time points; pre-steam (PS), 3 dapys post steaming (A), 37 days post steaming (D), 426 days post steaming (Z), and time not applicable (NA). [file Image_1.jpeg]

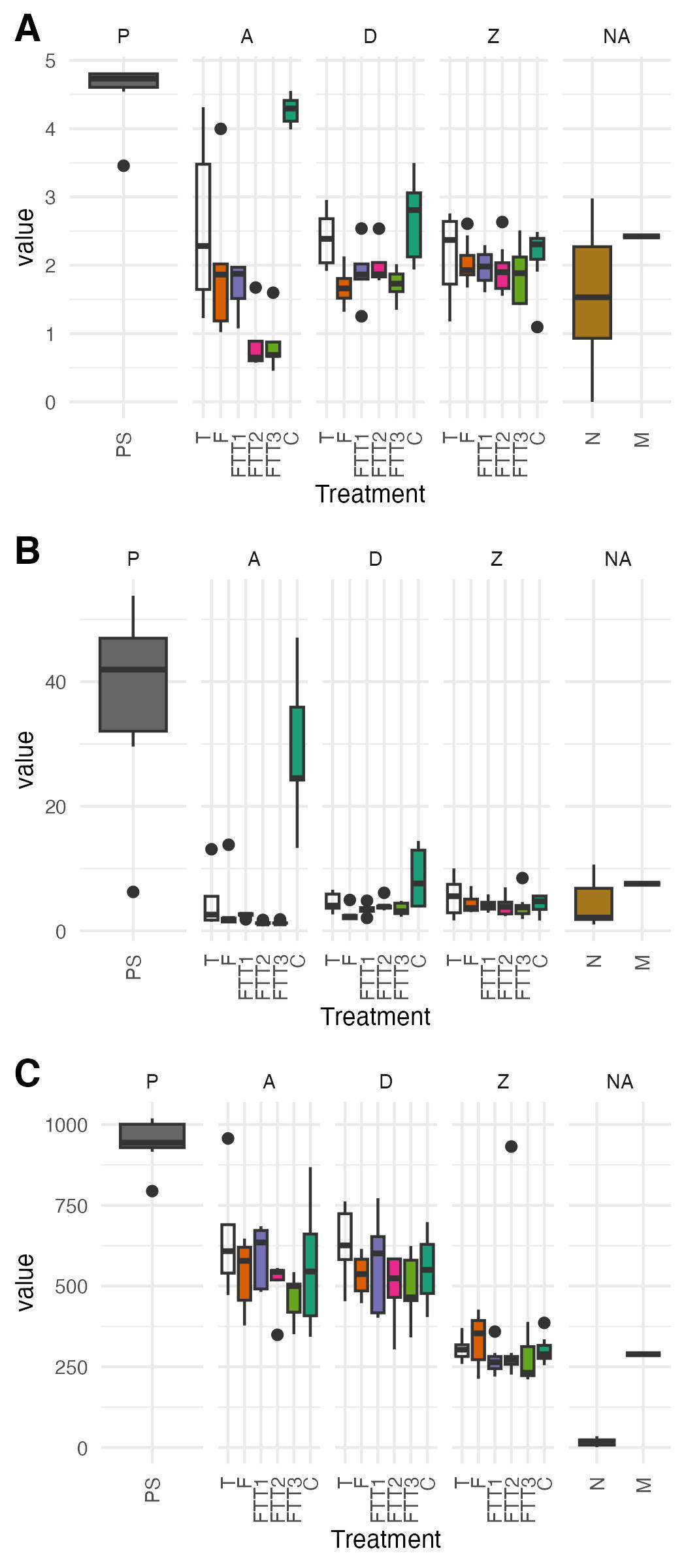

Supplement: Supplementary Figure 2 — The alpha diversity in each treatment was assessed at each time point by three diversity indexes: (A) Shannon (B) Inverse Simpson (C) Observed. The resulting data were presented as box plots with the index value on the y-axis and the treatments on the x-axis; pre-steam (PS), Trichoderma (T), Fusarium (F), Fusarium Trichoderma Time 1 (FTT1), Fusarium Trichoderma Time 2 (FTT2), Fusarium Trichoderma Time 3 (FTT3), control (C), negative control (C), mock community (M). The plot is faceted by the sampling time points; pre-steam (PS), 3 days post steaming (A), 37 days post steaming (D), 426 days post steaming (Z), and time not applicable (NA). [file Image_2.jpeg]

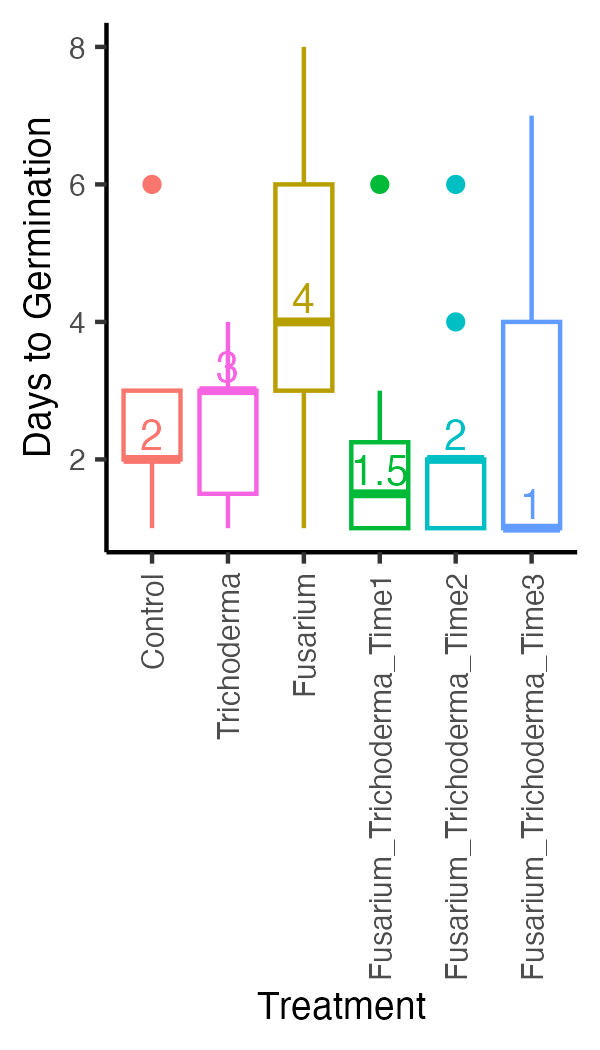

Supplement: Supplementary Figure 3 — After the final soil sample was collected at 426 days post-steaming, a susceptible variety of soybean (SC9277R) was planted in each pot. The pots were monitored daily and the days to germination were recorded. The data are presented as a box plot with treatment on the x-axis and the days to germination on the y-axis. No significant difference was found between treatments, however, the Fusarium treatment, all soybean seeds germinated 4 days later compared to the rest of the treatments. [file Image_3.jpeg]

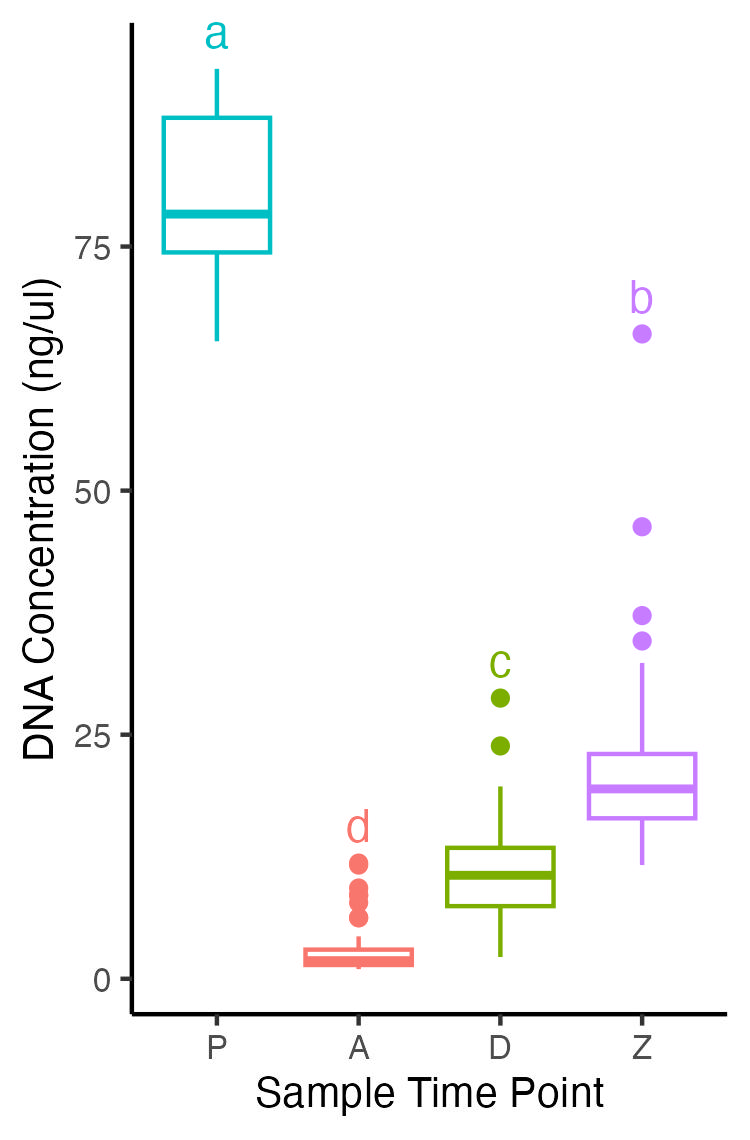

Supplement: Supplementary Figure 4 — After the DNA was extracted from each soil sample the DNA yield (ng/ml) was quantified. These data were analyzed by time point and were averaged across all treatments. The treatments were: pre-steamed soil (P), 3 days post-steam (A), 37 days post-steam (D), and 426 days post-steam (Z). Time point was found to be significant (p=2e-16). A post-hock Tukey Test confirmed that each time point to be significantly different from the others, as seen by the letters assigned. [file Image_4.jpeg]

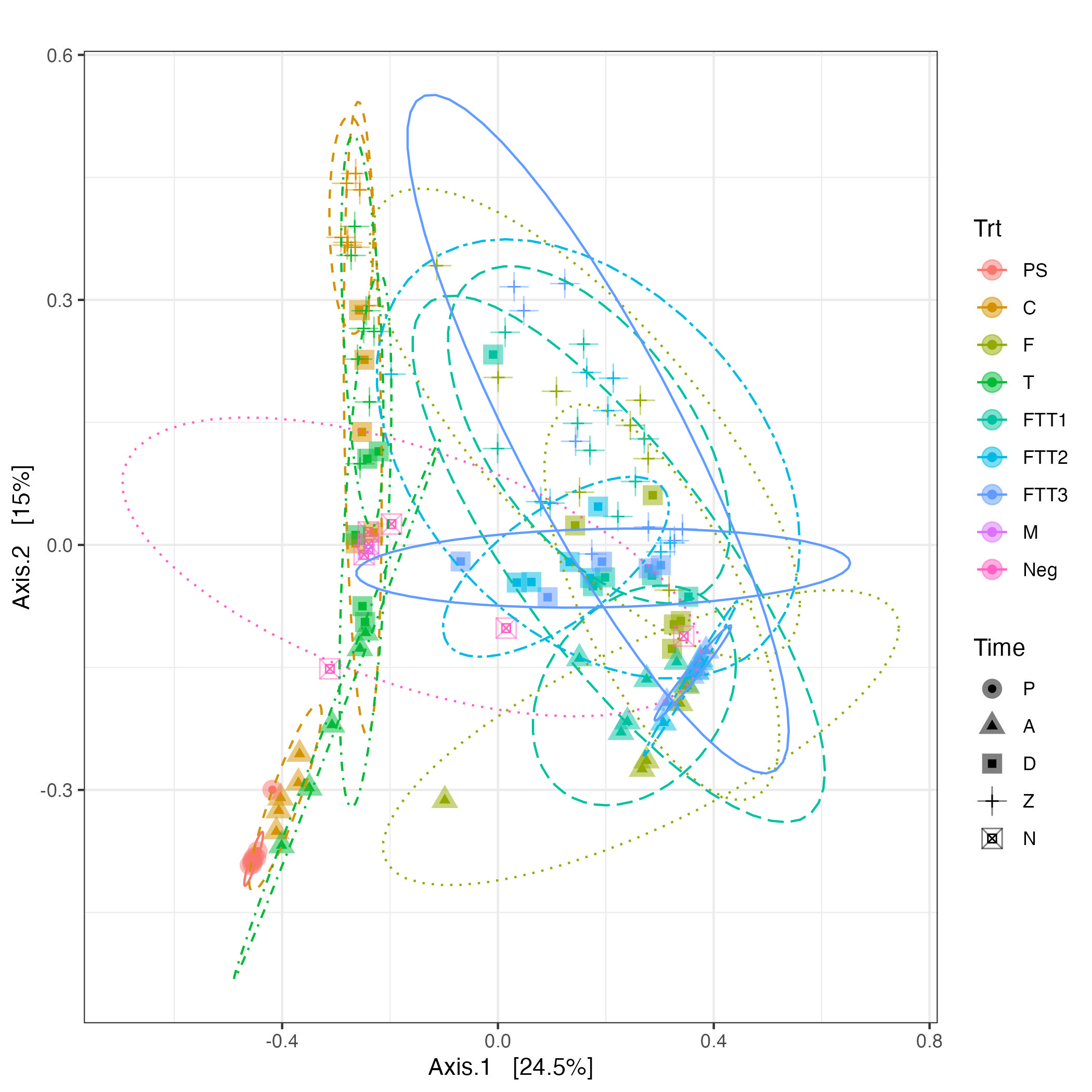

Supplement: Supplementary Figure 5 — A principal coordinate analysis (PCoA) with Bray-Curtis dissimilarity index was plotted for the six soil treatments; control (C), Fusarium (F), Trichoderma (T), Fusarium Trichoderma Time 1 (FTT1), Fusarium Trichoderma Time 2 (FTT2), and Fusarium Trichoderma Time 3 (FTT3). The pre-steamed soil (PS), the mock community (M), and the negative controls (N), are also included in the ordination. 95% confidence ellipses are drawn. Shapes (e.g., circles, triangles) delineated the time point that each sample was taken at. The time points were (P) pre-steam, (A) 3 days post steaming, (D) 37 days post steaming, (Z) 426 days post steaming, and (N) no applicable time point. As the time points progressed from A to Z, the samples for each treatment moved in the positive direction on Axis.2. Samples of treatments F, FTT1, FTT2, and FTT3, also moved in the negative direction on Axis.1 from time point A to Z. This resulted in the fungal communities within each treatment converging in their taxonomic similarity over time. [file Image_5.jpeg]
